# Response of *Thalassia Testudinum* Morphometry and Distribution to Environmental Drivers in a Pristine Tropical Lagoon

**DOI:** 10.1371/journal.pone.0164014

**Published:** 2016-10-13

**Authors:** Israel Medina-Gómez, Christopher J. Madden, Jorge Herrera-Silveira, Björn Kjerfve

**Affiliations:** 1 Departamento de Recursos del Mar, Laboratorio de Producción Primaria, CINVESTAV-IPN, Mérida, Yucatán, México; 2 South Florida Water Management District, West Palm Beach, Florida, United States of America; 3 American University of Sharjah, University City, Sharjah, United Arab Emirates; Universita degli Studi di Genova, ITALY

## Abstract

This study was undertaken to determine the relationships between the biomass, morphometry, and density of short shoots (SS) of the tropical seagrass *Thalassia testudinum* and the physical-environmental forcing in the region. Seasonal sampling surveys were undertaken four times in Bahia de la Ascension, a shallow estuary in the western Mexican Caribbean, to measure plant morphology and environmental variables. The estuary has a fresh water-influenced inner bay, a large central basin and a marine zone featuring a barrier reef at the seaward margin. Leaf size was positively correlated with increasing salinity, but total biomass was not, being similar across most of the sites. Aboveground biomass exhibited seasonal differences in dry and rainy seasons along the bay, most markedly in the brackish inner bay where an abrupt decline in biomass coincided with the rainy season. The relationship between nutrients and biomass indicates that the aboveground/belowground biomass ratio increases as nutrient availability increases. Areal cover was inversely correlated with SS density during both dry and rainy seasons. Maximum SS recruitment coincided with the rainy season. Peaks in SS density were recorded in the freshwater-influenced inner bay during an ENSO cold phase in 2007 (“La Niña”) which is associated with a wetter dry season and following a strong storm (Hurricane Dean). The onset of the rainy season influences both shoot density and *T*. *testudinum* biomass by controlling the freshwater input to the bay and thus, the system’s salinity gradient and external nutrients supply from the coastal wetland.

## Introduction

Seagrass communities constitute a major feature in submerged aquatic vegetation (SAV) in shallow marine waters around the globe [1]. These marine angiosperms provide important functions of sediment trapping and stabilization, wave energy dissipation [2], regulation of nutrient cycling [3], and they constitute important shelter and feeding habitat [4–6]. Seagrasses are keystone species of great importance to coastal communities and seagrass die-off events are associated with decreases in abundance of fish and benthic invertebrates [7].

Seagrass meadows of tropical latitudes have been shown to increase ecosystem connectivity via tidally-driven and nekton-facilitated organic material exchange between mangrove habitat and coral reefs [8]. They also export a portion of their organic production offshore, representing a source of material and energy to the marine shelf [9]. Such linkages among seagrasses and other habitats render them not only one of the most productive tropical marine communities in their own right, but also enhance the productivity of adjacent systems. Owing to their location in shallow nearshore sites prone to human development, these communities are vulnerable to cultural eutrophication. Subjected to nutrient enrichment they experience increases in light competition from macroalgae, phytoplankton and epiphytes resulting in reduced seagrass coverage and loss of ecosystem function [10,11].

The stenohaline seagrass *Thalassia testudinum* [12] is ubiquitous in bays and coastal lagoons of the Caribbean, as well as in shallow coastal waters of the marine shelf [13]. This species develops extensive meadows often in proximity to mangrove forests that might be seasonally influenced by runoff [14]. Individuals colonizing areas adjacent to tidal creeks have shown reduced total biomass and production, along with blade narrowing, suggesting the effects of osmotic stress from fresh water [15]. Seagrasses in karst environments such as the Yucatan peninsula, characterized by substrates of high porosity and permeability, are susceptible to subaqueous flows driven by freshwater infiltration from the groundwater network [16,17]. Thus, vegetation beds along nearshore locations may reflect some degree of stress particularly during wet seasons as a consequence of freshwater supply [18].

A mesocosm study indicated *T*. *testudinum* can survive reduced salinities for several weeks, although aboveground biomass tends to decline in lower salinities [19]. Thorhaug et al. [20] found physiological changes in the *T*. *testudinum* leaf in mesohaline conditions. Negative effects on photosynthetic efficiency was more marked in young individuals than in mature shoots [21].

The development of *T*. *testudinum* under suboptimal salinity conditions fostered by high freshwater input implies a tradeoff between a useful land-derived nutrient supply and environmental stress from low salinity conditions. This scenario may be enhanced due to climate change when global trends of multi-decadal warming are predicted to bring about higher ENSO occurrences under some continental-scale models [22]. Cold phase La Niña episodes have been associated with a wetter dry season in a shallow coastal bay of the western Caribbean and consequently greater freshwater influence in the system [23]. Such predictions of broad-scale climate change may threaten pristine seagrass meadows particularly in tropical coastal zones, highlighting the need for baseline information on the responses of SAV communities to its environmental setting.

This study assesses the development of *T*. *testudinum* along a salinity and nutrient gradient in a shallow coastal bay influenced by groundwater discharges in the western Caribbean. We address the range of morphometric traits, structural features, and short shoot densities exhibited by *T*. *testudinum* in a mangrove-fringed, groundwater-influenced system. We examine how total biomass in these lower salinity beds differs from those developing under optimum conditions at the seaward boundary of the system. We hypothesize that despite the greater nutrient subsidy provided in land runoff, plants exposed to mesohaline salinities will show reduced aboveground biomass, leaf size, and short-shoot density due to hyposalinity stress.

### Study Site

**Fig 1:**
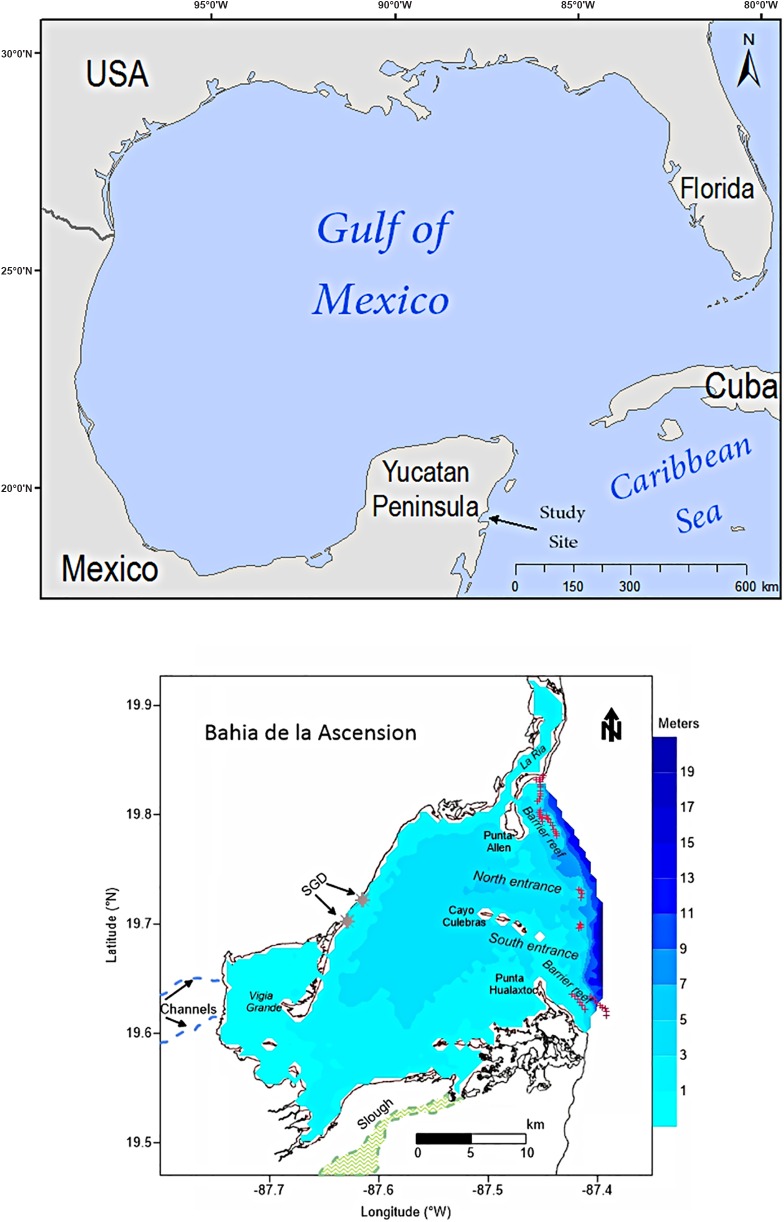
(above) Map of Gulf of Mexico showing the location of the study site (arrow). (below) Bahia de la Ascension in the Western Caribbean with bathymetry (meters). Main geomorphologic features within the system are shown, along with coral reef formation at the seaward boundary. Freshwater sources into the bay are depicted (SGD: submerged groundwater discharges).

Bahia de la Ascension (BA) is a shallow coastal bay occupying a flooded karst depression in the Sian Ka’an Biosphere Reserve (SKBR), eastern Yucatan Peninsula (YP) (Fig 1a). The system has a surface area of 580 km^2^ and a drainage basin of 1,200 km^2^. It is confined by two headlands, Punta Hualaxtoc to the south and Punta Allen to the north (Fig 1b). The latter is the location of a small fishermen’s village (469 total population) [24], the only populated site along the bay’s coastline. The valuable resources that Bahia de la Ascension provides to the local inhabitants, such as the traditional commercial spiny lobster fishery, recreational fishing operation, and burgeoning commercial activities associated with tourism and ecotourism, are tied directly and indirectly to the environmental well-being of the mangrove-seagrass-coral reef continuum.

The mean depth of the bay is 2.2 m, with a maximum of 6.8 m in the main, E-W oriented tidal channel located between Punta Allen and Cayo Culebras, a mangrove cay in the middle of the main bay-reef lagoon boundary. Tides are mixed semi-diurnal, featuring a micro-tidal regime [25]. The main axis of Bahia de la Ascension is oriented SW-NE with a semi-continuous coral barrier reef defining its seaward boundary (Fig 1b). This formation is a segment of the Mesoamerican barrier reef system (MBRS), the second largest reef system in the world, spanning four countries of the Western Caribbean: Mexico, Belize, Guatemala, and Honduras.

The region is influenced by marked seasonality of a dry season (March to June) and rainy season (July to October), and a winter season of episodic fronts when rainfall accounts for about 10% of the annual mean [26]. The Caribbean coast of the YP exhibits the highest occurrence of hurricanes in Mexico [27]. The low relief of the YP offers little resistance to hurricanes, which often traverse the entire Peninsula. Hurricane Dean, the most recent tropical cyclone to impact Bahia de la Ascension, made landfall on the Yucatan Peninsula as a category 5 storm on 21 August 2007 100 km south of Bahia de la Ascension. Maximum sustained winds at landfall were 277.8 km hr^-1^ [28].

The karstic soil of the low relief Yucatan precludes significant surface water flow. Rather, the bay receives a significant amount of freshwater through submerged springs groundwater drainage into the southwestern-most of the bay (Fig 1b). The widely distributed submerged aquatic vegetation is dominated by the seagrass species *T*. *testudinum*, which extends from the marine-influenced reef lagoon to the mesohaline environment at the inner-most portion of the bay. Because BA lies completely within the protected SKBR, it is relatively unimpacted by human activity and a suitable site for research addressing issues of connectivity to adjacent systems, and SAV responses to upstream sources and the agriculture and tourism development in and near the watershed.

## Materials and Methods

**Fig 2:**
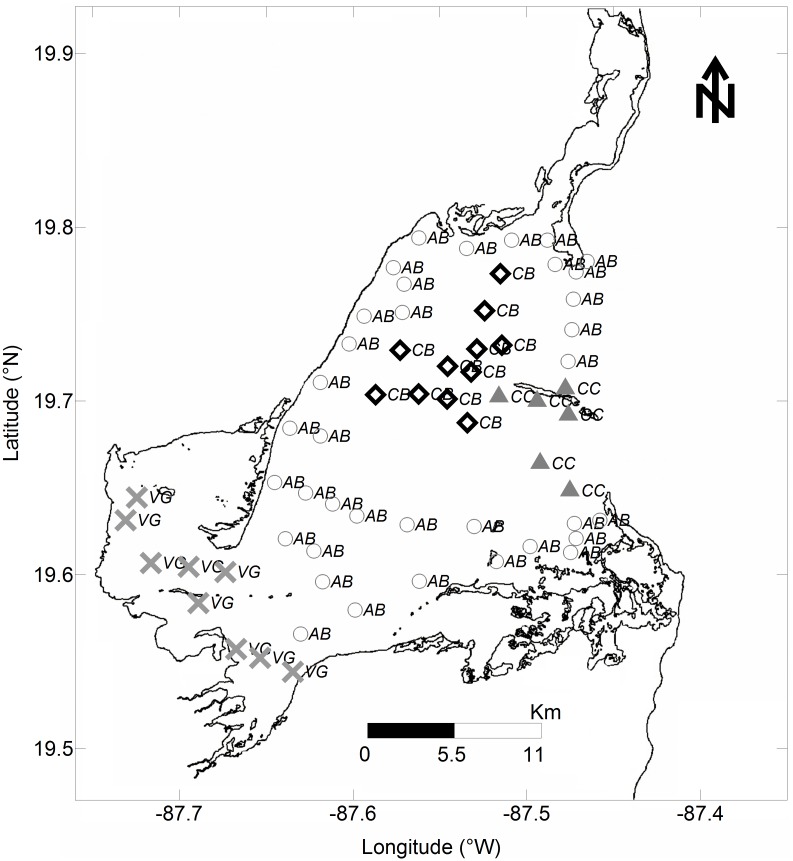
Study site showing sampling stations across three *Thalassia testudinum* beds referenced throughout the paper. Cayo Culebras (CC = ▲; n = 6), Central Basin (CB = ◊; n = 11), and Vigia Grande (VG = ×; n = 9). Stations where only abiotic parameters were collected are also shown (AB = ○; n = 36). Total number of sampling stations is 62.

Four sampling surveys of seagrasses and environmental variables were undertaken in BA during 2006 and 2007. Two seasonal surveys were carried out each year during the June dry season and October rainy season. Sixty-two stations (Fig 2) were sampled for hydrographic parameters, and at 26 stations plant morphometrics were recorded. While short-shoots density and abundance data were available both for 2006 and 2007 campaigns, biomass, leaf length and leaf width measurements corresponded only to 2006.

### Hydrographic variables

Conductivity-Temperature profiles were taken with a SeaCAT Profiler. Vertical stratification was generally not evident throughout the bay during these seasonal surveys, except for an ephemeral vertical structure confined in a narrow band near Punta Allen during the rainy season in 2007 [23]. Thus, water samples were collected at mid-depth at each station for further nutrients analysis. Sample preservation, processing, and determination of nitrite, nitrate, ammonium, soluble reactive silica (SRSi), and soluble reactive phosphorus (SRP) were performed spectrophotometrically according to Medina-Gómez and Herrera-Silveira [29]. Also, high resolution spatial sampling (<30 m horizontal distance) of relative fluorescence (rfu) for colored dissolved organic matter (CDOM) was performed using a continuous flow-through sampler system (Dataflow) [30] during the dry season survey in October 2007.

### Biological variables

Surveys were conducted at three sites (i.e., Vigia Grande, Central Basin, and Cayo Culebras) previously identified by Arellano-Méndez et al. [31] in a supervised classification of *T*. *testudinum* distribution in Bahia de la Ascension using a Landsat ETM^+^ image (taken on 21 April 2001). Vigia Grande (VG) is a shallow (sub-meter average depth) SW embayment featuring fine sand-silt sediments and receiving freshwater from small surface channels and groundwater discharges. The central basin (CB), 3 m average depth, is located at mid-bay and characterized by rocky bottom and coarse sandy cover. *T*. *testudinum* constitutes the dominant species at these two sites. Cayo Culebras (CC) is a small and shallow (<0.6 m) NW-SE oriented sandy cay with a well-developed mangrove cover (e.g. *Rhizophora mangle*, *Avicennia germinans*, and *Laguncularia racemosa*) contiguous to the reef lagoon. At this site *T*. *testudinum* co-occurs with *Halodule wrightii* in a mixed vegetation community protected from prevalent NE Trade winds.

A total of 26 stations (Vigia Grande = 9 st, Central Basin = 11 st, and Cayo Culebras = 6 st.) were selected from these optically-distinct seagrass zones, with the number of sampling stations reflecting the corresponding surface areas of the classes they represented (larger zones had larger *n*). This sampling scheme took advantage of the strong horizontal salinity gradient characterizing the system between the freshwater-influenced inner bay (southwest) and the marine-influenced seaward boundary (northeast) [23]. The resultant sampling station network is considered appropriate to characterize *T*. *testudinum* responsiveness to both nutrients and salinity variability as freshwater input correlates with nutrients supply in the Yucatan landscape [29].

Submerged aquatic vegetation (SAV) was surveyed using the Braun-Blanquet (B-B) technique for a rapid visual assessment of SAV cover [32]. Four replicate quadrats (0.25 m²) were sampled at each of the 26 stations using SCUBA. Cover values for all seagrass species were recorded, and a B-B score from 0–5 was assigned based on the estimated cover of each species [32]. Also, above and below ground biomass were collected using a PVC core (25 cm diameter). The collected material was cleaned and preserved in 10% formaldehyde for further processing in the laboratory. The collection of samples and treatment of biomass was undertaken according to the CARICOMP methods manual for seagrass communities [33].

Secchi depth exceeded bottom depth at all of the sampling stations (or equal to 0.5 m in the inner embayment “Vigia Grande”, 2.5 m in the bay’s Central Basin, and 1.0 m in the mangrove cay “Cayo Culebras” at the seaward boundary) and light was not considered limiting. During the first two surveys in 2006 plants were analyzed for epiphytes cover and encrusting algae was observed colonizing *T*. *testudinum* leaves in “Cayo Culebras” mangrove cay. However, preliminary analysis indicated an overall low biomass contribution by epiphytes. The epiphytic biota found in this location is similar to that previously described elsewhere in the Caribbean under pristine reef environments where encrusting coralline algae are dominant [34]. Thus, we found no sign of anomalous epiphytism in these seagrass sites studied.

### Statistical analyses

Vegetation response to seasonal environmental factors was assessed by comparing the seasonal variability of *T*. *testudinum* metrics at three seagrass meadows along the SW-NE bay’s axis: “Vigia Grande,” the central bay, and “Cayo Culebras.” ANOVA’s for the hydrographic and water quality parameters allowed testing the null hypothesis that means are statistically the same. P-values for the F-test were calculated and inspected for significant differences (p-value < 0.05) among the means of a given parameter corresponding to the distinct levels of a “treatment” at the 95.0% confidence level.

The seagrass parameters were first examined to determine whether they could be adequately modeled by a normal distribution (Chi-square and Shapiro-Wilks tests). P-values less than 0.01 for these tests indicated the data were not normally distributed with 99% confidence. Because preserving the original data to facilitate interpretation of the relationship between variables was a priority, no further transformation was applied to the biological dataset to force a normal-like distribution. Thus, Kruskal-Wallis test was utilized instead as an alternative non-parametric analysis of variance by ranks to test the null hypothesis that the medians of the distinct seagrass parameters are the same. This analysis does not require equal numbers of observations in each group [35]. P-values for the Kruskal-Wallis test statistic H were assessed for statistically significant difference amongst the medians at the 95.0% confidence level. Statistical analyses were performed utilizing STATGRAPHICS Centurion XV, Ver. 15.1.02 (StatPoint, Inc. 1982–2006).

A Principal Component Analysis (PCA) was applied per survey (2006–2007) to the physical-chemical parameters (temperature, salinity, dissolved oxygen) and dissolved inorganic nutrients (NO_2_^-^, NO_3_^-^, NH_4_^+^, SRP, SRSi) to identify environmental gradients and key variables controlling them.

Also, a Detrended Correspondence Analysis was implemented to assess the size of the gradient in the seagrass vegetation variables and inspect whether a multivariate Redundancy Analysis (RDA) or a Canonical Correspondence Analysis (CCA) was the most suitable canonical analysis to analyze relevant relationships between our environmental and biologic variables (S1 Fig and S1 Table). The results of such a preliminary analysis led us to apply a RDA separately per year (2006, 2007) and period (dry and rainy) to elucidate the proportion of total variance in *T*. *testudinum* variables (e.g. demographic, structural, and morphometric) explained by or predicted from such physical-environmental gradients in the system [36]. These analyses were undertaken using only the variables scoring highest coefficients in the annual PCA’s. The statistical significance of the RDA model was tested using a Monte Carlo randomization test.

Finally, using a mixed effect model analysis carried out in *R* environment (RStudio Inc., Ver. 0.98.976, 2009–2013), we examined the statistical interactions between *T*. *testudinum* and the physical-environmental variability, by investigating the influences (both independently and combined) of two factors: period of the year (with two levels—dry and rainy), and salinity gradient (with three treatments—brackish, mixed, oceanic) on the biotic data variance.

No specific permissions were required for carrying out these activities in this study site. The field studies did not involve endangered or protected species.

## Results

**Fig 3:**
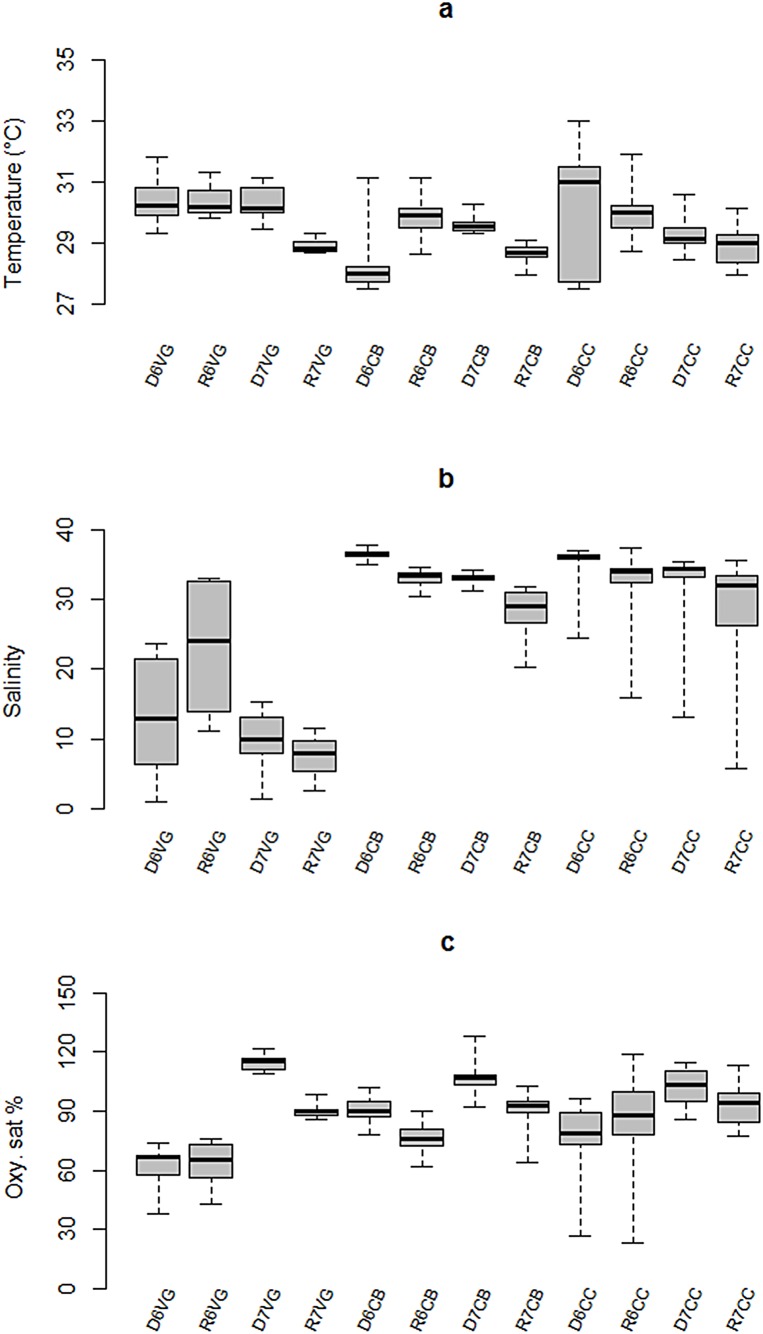
Seasonal variability of hydrographic properties in Bahia de la Ascension: (a) temperature, (b) salinity, (c) dissolved oxygen. Median is the horizontal line into the box, 25th and 75th percentiles are the top and bottom of the box, while the 5th and 95th are located on the tips of the whiskers. The labels on x-axis: first letters represent the climatic season (*D*ry, or *R*ainy); second number designs the year of sampling campaign (*6* for 2006 and *7* for 2007); following letters are the sampling site (Vigia Grande, Central Basin, and Cayo Culebras).

Water temperature was generally less variable in interior bay than either in the Central Basin, or in the marine end member, with statistically lower values during rainy season 2007 across the bay (F = 7.66, p = 0.0, DoF = 11). During the 2006 dry season, the lowest mean temperature of the study was observed in the central bay, and highest values were observed at the seaward boundary (Fig 3a). In general, salinities during the 2007 survey were lower than in 2006 at all sites in rainy season 2007. Lowest salinities were consistently observed in the SW inner bay (Fig 3b). During the 2006 survey, average dissolved oxygen was significantly lower at the brackish-influenced, inner bay than in the central basin and marine zone. Mean DO in the 2007 dry season was significantly higher at all sites than in the rainy season (F = 17.02, p = 0.0, DoF = 11) (Fig 3c).

### Nitrogen

**Fig 4:**
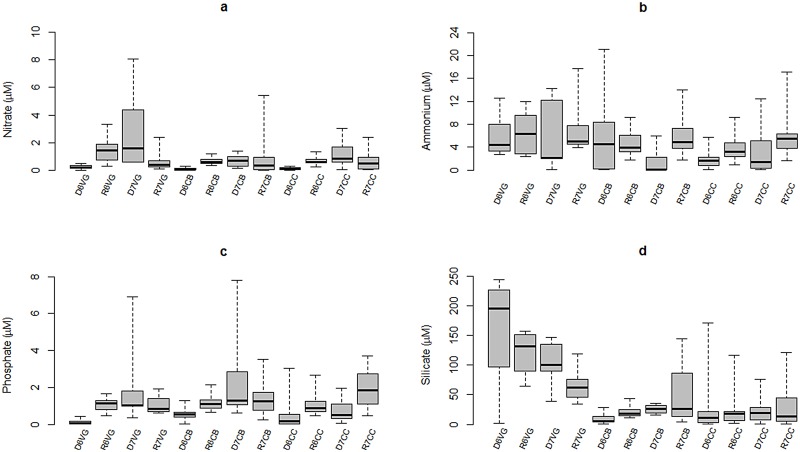
Seasonal variability of inorganic nutrients in Bahia de la Ascension: (a) nitrate, (b) ammonium, (c) phosphate, (d) silicate. Median is the horizontal line into the box, 25th and 75th percentiles are the top and bottom of the box, while the 5th and 95th are located on the tips of the whiskers. The labels on x-axis: first letters represent the climatic season (*D*ry, or *R*ainy); second number designs the year of sampling campaign (*6* for 2006 and *7* for 2007); following letters are the sampling site (Vigia Grande, Central Basin, and Cayo Culebras).

**Fig 5:**
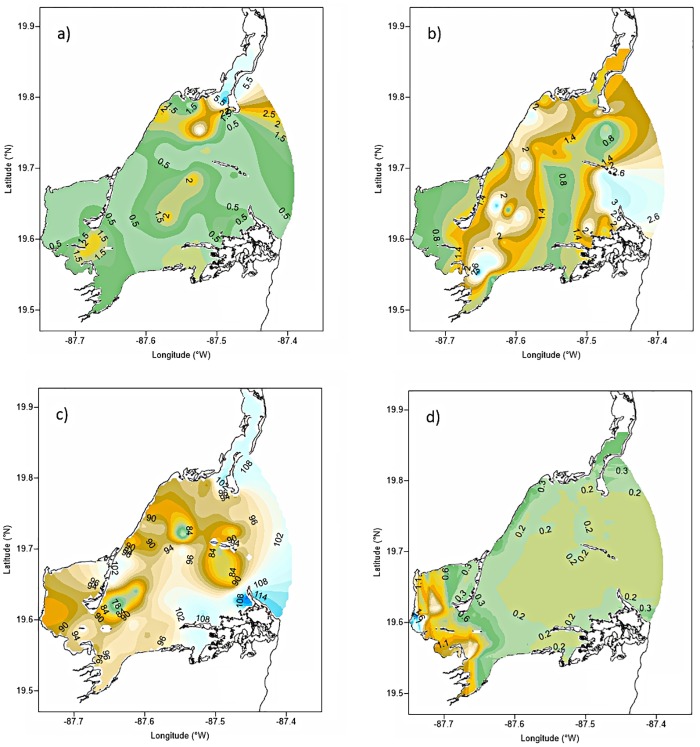
Spatial distribution of water quality parameters in Bahia de la Ascension. (a) nitrate (μM), (b) phosphate (μM), (c) dissolved oxygen (saturation percentage), and (d) colored dissolved organic matter—CDOM (mg L^-1^). All spatial patterns correspond to rainy season 2007, except for CDOM corresponding to dry season 2007.

Nitrate concentrations in 2006 were significantly higher during the rainy season relative to the dry season (F = 7.30, p = 0.0, DoF = 11), but this pattern was reversed in 2007, when all sites showed lower nitrate concentrations during the rainy season. This condition was statistically significant only in the inner-most embayment (p-value of the F-Test << 0.05) (Fig 4a). Spatially, highest nitrate concentrations were recorded at nearby Punta Allen, while a drop in concentrations was observed in the inner-most bay (<1 μM) (Fig 5a). Higher and more variable NH_4_^+^ concentrations were observed in the inner zone compared to the central basin and the seaward mangrove cay regardless of the season or year (Fig 4b). Higher NO_3_^-^ concentrations and variability was observed in the inner bay during the dry season 2007 compared to the other sites during the study.

### Phosphate

Mean phosphate concentrations in dry season 2006 were statistically lower than those recorded in rainy season for the three sites (F = 7.20, p = 0.0, DoF = 11) (Fig 4c). This relationship changed in 2007 for the inner-most SW embayment and the central basin, when highest and more variable PO_4_^-3^ concentrations were recorded during the dry season (6.9 μM and 7.8 μM, respectively). Such differences, however, were not statistically significant (p-value of the F-Test > 0.05). Although PO_4_^-3^ values above 1 μM pervaded the whole central basin, peak concentrations corresponded to the southern headland “Punta Hualaxtoc”, which harbors abundant waterfowl (Figs 1b and 5b). An increasing phosphate gradient occurred towards the marine inlet there, with values up to 3 μM.

### Silicate

**Fig 6:**
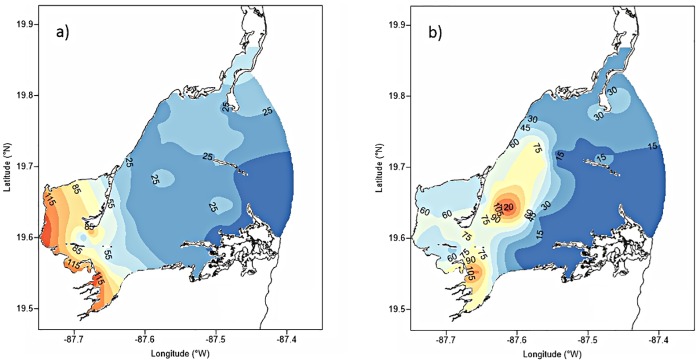
Spatial distribution of SRSi (μM) in Bahia de la Ascension during 2007. (a) dry season, (b) rainy season.

SRSi exhibited three patterns of variability in the sites studied. Silicate concentrations in the inner-most bay were substantially higher than those in the central basin or the mangrove cay site (F = 20.66, p = 0.0, DoF = 11). The highest average SRSi value was recorded in the inner-most embayment during the dry season 2006, reflecting a decreasing trend seaward (Fig 4d). In the central basin, mean SRSi concentrations depicted an opposite seasonal pattern of the inner subsystem, with maximum values during the rainy season 2007. The spatial distribution of SRSi concentrations in 2007 showed an overall gradient increasing toward the inner bay during both the dry and rainy seasons, although featuring highest values nearby the continental margin during rainy season, coinciding with groundwater discharges (Fig 6).

### Colored dissolved organic matter

The CDOM depicted a SW-NE spatial gradient across the bay in the dry season 2006 (the only available survey for this parameter). CDOM showed peak values up to 2.78 mg L^-1^ in the proximity of a surface stream emanating from the mangrove forest into the SW embayment, and lowest concentrations of 0.11 mg L^-1^ throughout the central basin and seaward boundary (Fig 5d). No data of CDOM is available for rainy season 2007 due to malfunctioning of the sensor.

### Biological variables

#### Biomass

The BA submerged aquatic vegetation community included several species, each showing a distinctive spatial pattern distribution across the bay. *Syringodium filiforme* and macroalgae as *Udotea* spp., *Penicillium* spp., and *Halophyla* spp. occurred in the reef lagoon-influenced open entrance. *Halodule wrightii* was interspersed with *T*. *testudinum* windward the mangrove cay “Cayo Culebras” forming dense mixed vegetation beds that also harbored *Batophora oerstedii* in the rainy season. *Ruppia maritima* was observed in the inner-most bay only during the rainy season. *T*. *testudinum* was present throughout the system, the dominant species in the community.

**Fig 7:**
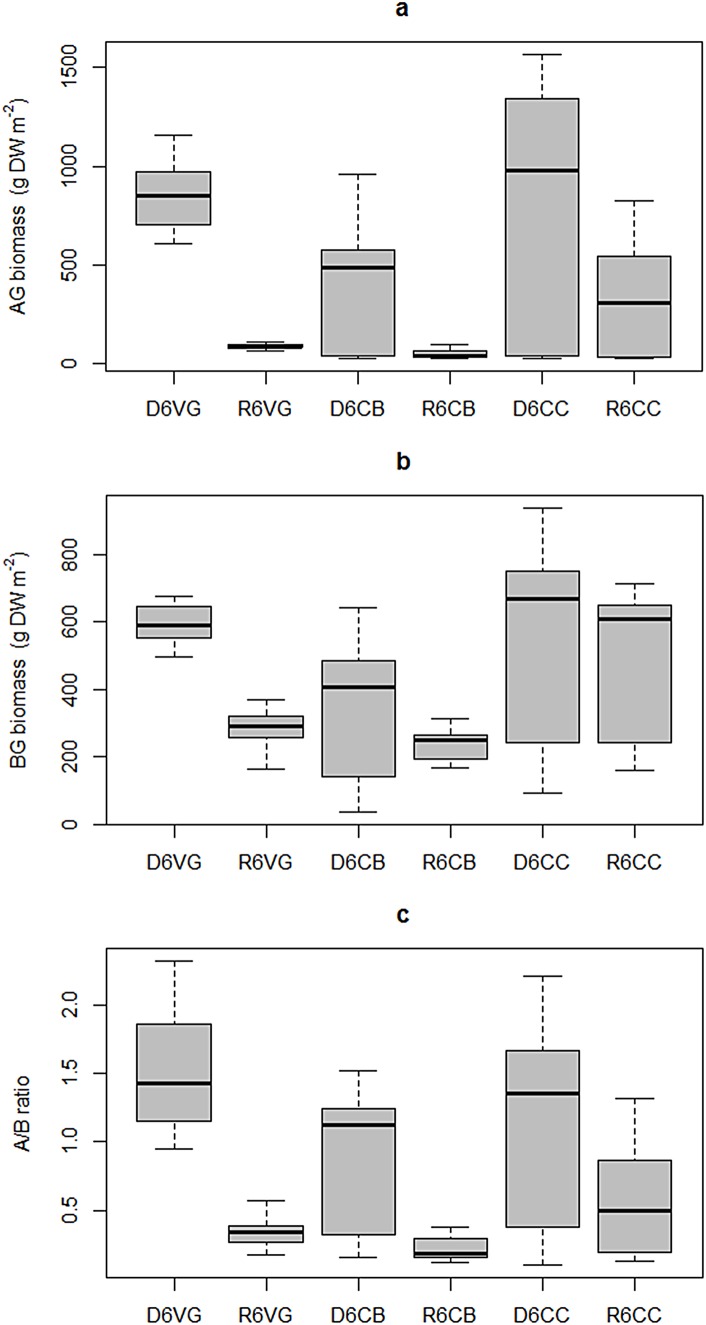
Intra-annual variability of *T*. *testudinum*. Aboveground biomass (a), belowground biomass (b), and aboveground/belowground ratio (c) in Bahia de la Ascension during 2006. Median is the horizontal line into the box, 25th and 75th percentiles are the top and bottom of the box, while the 5th and 95th are located on the tips of the whiskers. The labels on x-axis: first letters represent the climatic season (*D*ry, or *R*ainy); second number designs the year of sampling campaign (*6* for 2006 and *7* for 2007); following letters are the sampling site (Vigia Grande, Central Basin, and Cayo Culebras).

Significantly lower *T*. *testudinum* aboveground biomass was recorded in the rainy season relative to dry season for all three zones (Kruskal-Wallis test; H = 34.98, p = 1.52 x10^-6^, DoF = 5), with a larger seasonal difference in the inner bay (SW embayment). The *T*. *testudinum* bed at the seaward zone in the inlet showed both the highest median aboveground biomass (980 g DW m^-2^) and the greatest variability (range of 1,542.9 g DW m^-2^) during the dry season (Fig 7a). The central basin had the lowest biomass for both seasons. Also, no significant differences were detected between the aboveground biomass windward the mangrove cay and the SW subsystem during the dry season, even though biomass values were less scattered around the median in the inner subsystem (median of 853.7 g DW m^-2^ and range of 544.5 g DW m^-2^).

Belowground biomass exhibited a pattern similar to the aboveground component, overall lower values in the rainy season than in the dry season, and a strong seasonal variation in the inner bay and central basin (H = 30.09, p = 1.41 x10^-5^, DoF = 5). However, no significant seasonal differences of belowground biomass occurred in the mangrove cay at the inlets zone, which also recorded the highest median biomass of 667.56 g DW m^-2^ in the dry seasons (Fig 7b).

#### Aboveground/Belowground biomass ratio (AG/BG)

The AG/BG ratio decreased from the dry season to the rainy season, featuring ratios >1 only during the former. The seasonal AG/BG differences were statistically significant at the 95% confidence level for each location (Kruskal-Wallis test; H = 43.57, p = 2.83 x10^-8^, DoF = 5) and this pattern was more prominent both in the inner embayment and the central bay. The highest average AG/BG ratio during dry season was recorded in the inner embayment (1.51) and in the bed near the mangrove cay (0.57) during the rainy season. Overall, the variability within zones increased from the bay’s interior towards the inlet zone (Fig 7c).

#### Leaf length and width

**Fig 8:**
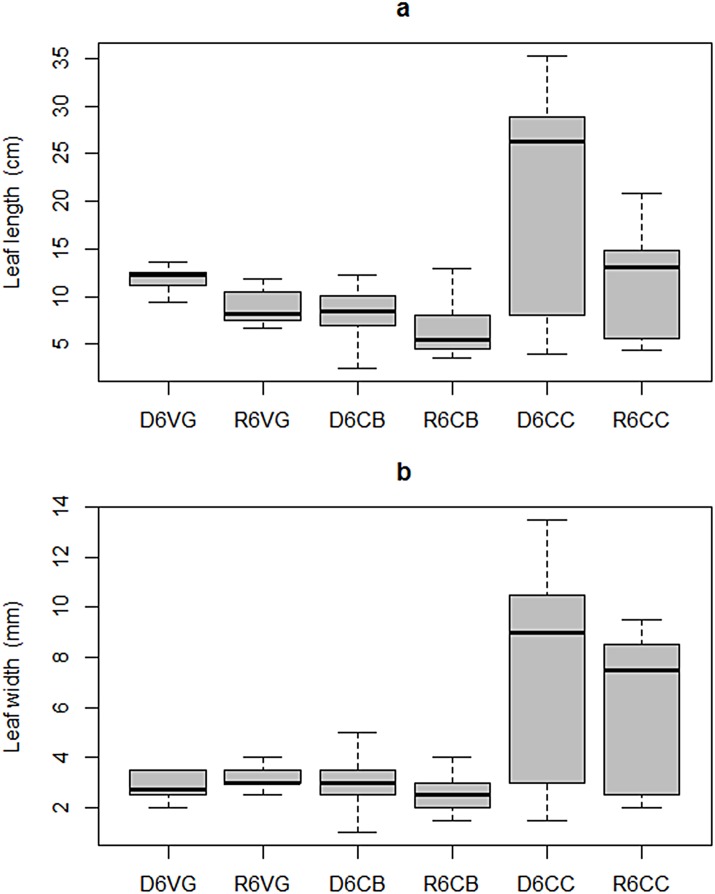
Intra-annual variability of *T*. *testudinum* leaf morphometry in Bahia de la Ascension during 2006: (a) length, (b) width. Median is the horizontal line into the box, 25th and 75th percentiles are the top and bottom of the box, while the 5th and 95th are located on the tips of the whiskers. The labels on x-axis: first letters represent the climatic season (*D*ry, or *R*ainy); second number designs the year of sampling campaign (*6* for 2006 and *7* for 2007); following letters are the sampling site (Vigia Grande, Central Basin, and Cayo Culebras).

The length of *T*. *testudinum* leaves had a clear seasonal variability, characterized by statistically significant shorter blades in the rainy season across the bay (Kruskal-Wallis test; H = 31.32, p = 8.10 x10^-6^, DoF = 5). This intra-annual pattern was relatively more apparent in the inner bay zone than anywhere else. Also during rainy period an asymmetrical distribution of leaf length with a long tail to the right (skewness = 1.21) was observed in the central basin, which is consistent with atypical long blades. The mangrove cay exhibited the longest blades, with median length of 26.3 cm and 13.1 cm in dry and rainy seasons, respectively. Leaf length in the mangrove cay was characterized by high variability, yielding coefficients of variation of 55% and 45% in dry and rainy seasons, respectively (Fig 8a).

Significantly larger leaf width (H = 28.61, p = 2.76 x10^-5^, DoF = 5) and consistently higher variability (CV = 58% and 47% in dry and rainy seasons, respectively) was observed near the mangrove cay than in the inner-most embayment, or in the main basin during both seasons (Fig 8b). Neither the central basin nor the inner bay sites exhibited seasonal blade width differences.

#### Abundance and Shoot density

**Fig 9:**
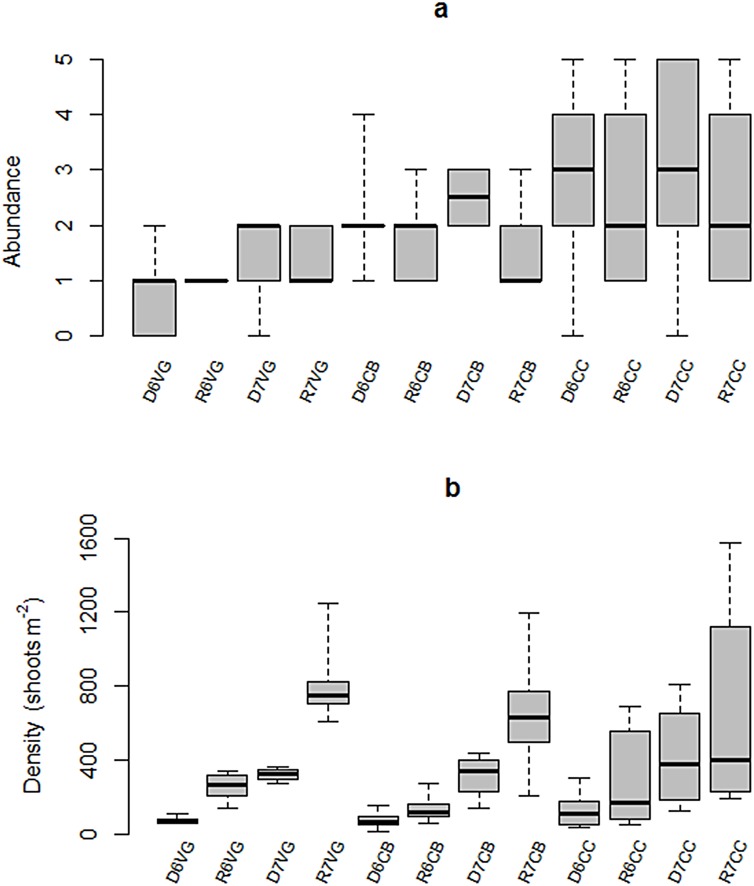
Seasonal variability of *T*. *testudinum* (a) abundance (given in Braun-Blanquet scores) and (b) shoot density in Bahia de la Ascension during 2006–2007. Median is the horizontal line into the box, 25th and 75th percentiles are the top and bottom of the box, while the 5th and 95th are located on the tips of the whiskers. The labels on x-axis: first letters represent the climatic season (*D*ry, or *R*ainy); second number designs the year of sampling campaign (*6* for 2006 and *7* for 2007); following letters are the sampling site (Vigia Grande, Central Basin, and Cayo Culebras).

Spatially, the median abundance of *T*. *testudinum* was lower in the inner-most zone of the bay and increased toward the seaward section (Fig 9a). Peak abundance occurred during the dry season and declined in the rainy season. This seasonality was strongest both in the central basin and mangrove cay.

Shoot density increased both seasonally and inter-annually during the two years of the study: increasing shoot densities during rainy season relative to those recorded in dry season, and higher shoot density during 2007 with respect to the preceding year. The Kruskal-Wallis Test results indicate that differences (e.g., intra-annual and interannual) in the bay’s interior and central basin are statistically significant at the 95% confidence level (H = 103.37, p = 0.0, DoF = 12).

The pattern was more evident in the inner bay, which exhibited a relatively low median density of shoots in the 2006 dry season (64 m^-2^), intermediate densities in the rainy 2006 season and dry 2007 season (261 and 323 shoots m^-2^, respectively; not significantly different), and peak, statistically significant median shoot densities (748 shoots m^-2^) during the 2007 rainy season (Fig 9b). Regardless of the large variability observed in the *T*. *testudinum* inhabiting mangrove cay in the inlet zone, no significant differences in shoot density were observed among samplings (Fig 9b).

### Statistical analyses

The Principal Component Analysis (PCA) integrating hydrographic and inorganic nutrients data accounted for 54.2% and 74% of the total variance during the 2006 (two components) and 2007 (three components) analyses, respectively (Table 1). During the 2006 survey, the principal component, PC-1, explained the opposite fluctuations between salinity and silicate, defining the groundwater discharge gradient. The PC-2 described the joint variability of nitrate and phosphate. Since concentrations of these nutrients are influenced by the assimilation in primary production, this relationship was synthesized as the production component.

**Table 1:** Principal Component Analysis (PCA) in Bahia de la Ascension during two years. Only eigenvalues >1 were kept. Percentage of variance per component and accumulated variance for all components are shown.

Survey	Eigenvalue	Key variables	Component name	%variance	Cum. %
2006	I: 2.94	Salinity, SRSi	*Groundwater input*	36.6	36.6
	II: 1.40	PO_4_^-3^, NO_3_^-^	*Primary production*	17.6	54.2
2007	I: 2.50	Salinity, SRSi	*Groundwater input*	31.0	31.0
	II: 2.04	NH_4_^+^, O_2_%	*Remineralization*	26.0	57.0
	III: 1.39	PO_4_^-3^, NO_3_^-^	*Primary production*	17.0	74.0

During the 2007 analysis, the first PC was also defined by the silicate and salinity gradient, accounting for by the groundwater input component. The concurrent dynamics of the oxygen saturation percent and ammonium concentrations (varying in the opposite direction along the ordination axis) in the second PC may indicate this component captured the biogeochemical decomposition of organic material into the oxidized stratum of sediments and thus, PC-2 was named the remineralization gradient. Again, nitrate and phosphate co-variation in PC-3 during 2007 suggests it may be capturing the gradient of nutrient uptake for primary production (production component).

**Fig 10:**
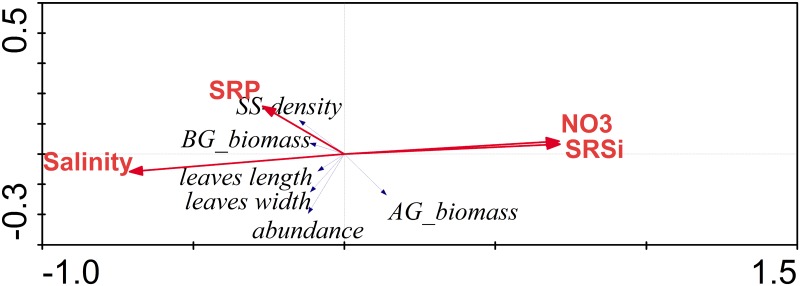
Triplot illustrating the RDA results during dry season 2006 in Bahia de la Ascension. First two canonical axes are shown.

The RDA applied on the dry season 2006 dataset was the only statistically significant analysis as supported by the Monte Carlo test (999 permutations under the unrestricted model; Table 2). The *T*. *testudinum* above ground biomass and short-shoot density varied inversely between them. These variables were influenced by the nitrate and silicate gradient (first axis; Fig 10). The second axis was explained by the salinity and phosphate gradient, with *T*. *testudinum* abundance aligning over it.

**Table 2:** Redundancy Analysis (RDA) per period (dry and rainy) during two years in Bahia de la Ascension. Eingenvalues and cumulative variance accounted by the first two axes into the model, as well as percentage of the whole variance (i.e. displayed by all the original variables) explained by the first two canonical axes computed in the RDA model are presented. Monte Carlo test results after 999 permutations under the reduced model are indicated (significant analyses are marked with an asterisk).

Analysis/season	Redundancy Analysis			Test of Significance	
	Eigen. axes I & II	% Cum. var. axes I & II	% total expl. Var.	F ratio	p-value
Dry-2006	0.153; 0.008	99.7	20	2.26	0.04*
Rainy-2006	0.143; 0.038	84.7	22	5.54	>0.05
Dry-2007	0.248; 0.102	78.1	50	2.86	>0.05
Rainy-2007	0.333; 0.077	95.6	43	7.01	>0.05

The mixed model analysis show that both components (above and belowground) of *T*. *testudinum* biomass were significantly different between climate periods, with relatively lower biomass in the rainy season compared to the dry season, and under mixed salinity condition compared to brackish environment. Also, high biomasses in the central basin and the seaward boundary depend on the influence (i.e. interaction) of the rainy season over these salinities (Table 3). Blade morphometry was statistically higher during the dry period and under marine-influenced salinities. *T*. *testudinum* leaves were significantly shorter in sites influenced by variable salinities, and individuals growing under marine salinity have shorter leaves when these sites are influenced by the rainy period. Short-shoots density was positively influenced by the rainy period while salinity treatments did not exert significant effects on its variability. Finally, abundance did show a significant increasing trend with the salinity treatments, but not with period of the year (Table 3).

**Table 3:** Mixed effect model fitted for each *Thalassia testudinum* response variable.

**A**						
**Dependent variable**	**AG biomass**	**SE**	**BG biomass**	**SE**	**Leaf length**	**SE**
Constant (Intercept)	870.999***	103.909	590.232***	58.586	11.890***	1.85
Period_rainy	-782.78***	146.949	-312.01***	82.853	-3.17	2.617
Mixed_salinity	-524.50***	126.248	-274.75***	71.181	-3.919*	2.248
Oceanic_salinity	-71.377	126.248	-34.792	71.181	8.491***	2.248
Period_rainy:Mixed_salinity	488.668***	178.541	230.968**	100.665	1.737	3.179
Period_rainy:Oceanic_salinity	319.271*	178.541	237.740**	100.665	-5.773*	3.179
R^2^	0.466		0.359		0.428	
F Statistic	17.092***		10.955***		14.669***	
**B**						
**Dependent variable**	**Leaf width**	**SE**	**Abundance**	**SE**	**SS-Density**	**SE**
Constant (Intercept)	2.800***	0.748	1.000***	0.31	142.643*	74.65
Period_rainy	0.45	1.058	0.154	0.44	260.203**	107.58
Mixed_salinity	0.152	0.909	1.120***	0.38	-31.923	93.24
Oceanic_salinity	4.533***	0.909	1.800***	0.37	78.89	90.40
Period_rainy:Mixed_salinity	-0.879	1.286	-0.693	0.54	-63.31	131.19
Period_rainy:Oceanic_salinity	-1.807	1.286	-0.141	0.53	-87.362	128.89
R^2^	0.415		0.27		0.135	
F Statistic	13.912***		10.283***		4.356***	

Two factors were used: period and salinity, with two (dry and rainy) and three (brackish, mixed, and oceanic) levels for each one of them, respectively. Also their statistical interactions were examined. Estimates (Standard error) with significant predictors are marked with asterisks (*p<0.1; **p<0.05; ***p<0.01).

## Discussion

The tropical seagrass *T*. *testudinum* displayed site-specific differences in biomass, leaf size, shoot density and abundance along salinity and nutrient gradients in Bahia de la Ascension (BA). This species developed across a wide range of salinity levels of this shallow coastal bay, from marine to mesohaline (salinity <16), yielding in this latter a peak total biomass of 1,800 g DW m^-2^ in the dry season. The relationship of biomass and blade morphometry to environmental variability was particularly strong in the brackish inner bay where low salinity likely prevents this population from attaining higher biomass levels as those observed in *T*. *testudinum* beds growing seaward.

Irlandi et al. [18] found significantly higher biomass in *T*. *testudinum* beds exposed to low freshwater influence than those developing in sites with abundant freshwater inflow. Also, higher short shoot survival rate has been reported in *T*. *testudinum* meadows inhabiting sites with low energy exposure in the Caribbean such as back-reef zones while lower survival is observed in energy intensive area in the reef lagoon and along the coastal edge [37]. However, *T*. *testudinum* stands growing in a shallow shoreface of the Mexican Caribbean, fronted by a reef edge which reduces wave energy, may attain considerable shoot densities provided that any major high-impact, low-frequency perturbation (category-5 hurricane) occurs in the region during a period long enough [38].

*T*. *testudinum* had the most developed stand at Cayo Culebras (CC), in a site protected by a mangrove cay seaward in BA. Proximity of CC to the reef lagoon and the open geomorphology at the inlet zone promotes a full marine salinity at that meadow [23]. Both empirical and modeling results suggest that growth rates in the bay are significantly elevated at oceanic salinities [17]. Consequently, the abundant vegetation bed observed in CC may be reflecting improved growth rates by this species under marine-influenced conditions and narrow salinity variability characterizing this area [23].

Also, this species can exhibit strong seasonal patterns of biomass in sub-tropical oligotrophic systems as in Florida Bay, documenting levels up to three-fold higher in summer than in winter [18]. Koltes et al. [39] reported biomass of 4,116 ± 681 g DW m^-2^ in summer in Twin Cays, Belize, about 300 km south of Bahia de la Ascension, one of the highest biomass measurements for this species globally and greater than the average biomass recorded in BA during late rainy season (956.2 ± 407 g DW m^-2^). As in BA, the Belize meadow is bounded by mangroves and seagrass biomass may also be supported by nutrient outwelling from there. However, the Belize location lacks significant freshwater input, contrasting with the inner-most section of BA, placed at the lower reaches of the Sian Ka’an coastal wetlands and influenced by lower salinities, high inorganic nutrient, and CDOM load (Fig 5).

Bay-wide median shoot density of 592 m^-2^ in the BA study site is well below that reported for a shoreline *T*. *testudinum* bed in the Mexican Caribbean, and a stand in Carrie Bow Cay in Belize, although within the range observed in a large hypersaline system of the western Gulf of Mexico (Table 4). However, average shoot density in BA is greater than densities recorded at distinct sites across the Caribbean (Table 4). Unusual high *T*. *testudinum* shoot densities recorded in the freshwater-influenced bay’s interior during the 2007 rainy season (maximum: 1,250 shoots m^-2^) is comparable to that reported by Koltes et al. [39] for *T*. *testudinum* across a shallow shelf in the western Caribbean (960 ± 250 m^-2^) adjacent to mangrove vegetation.

**Table 4:** Average *Thalassia testudinum* biomasses (g DW m^-2^) and short shoot densities (m^-2^) in shallow ecosystems of the Gulf of Mexico and the Caribbean Sea. Information drawn from [40], except for [38], [4,1], and [42].

Site	Total biomass	Shoot density	Authors
Bahia de la Ascension, Mexico	941 ± 675	592 ± 361	Current study
Puerto Morelos, Mexico (shoreline SAV)	—	1,678 ± 75.6	Enriquez and Pantoja-Reyes [38]
Carrie Bow Cay, Belize	4,116 ± 681	960 ± 250	Koltes et al. [39]
Florida Bay, U.S.A.	48.2	565.7	Hall et al. [41]
Lower Laguna Madre, U.S.A.	750–1,500	700–2,200	Kaldy and Dunton [42]
Cayo Coco, Cuba	931–2,396	755	Alcolado et al. [43]
Discovery bay, Jamaica	1,045.1	—	Gayle and Woodley [44]
Puerto Morelos, Mexico (reef lagoon SAV)	1,219.7	468	Ruiz-Renteria et al. [45]

A cold-phase ENSO, La Niña, event [46] and a category-5 hurricane converged on this region during 2007, and coincidently a dramatic increase in shoot number was recorded. The seeming influence of rainfall on short-shoot density reported by the mixed effect model (significant statistical interactions indicated with asterisks in Table 3) may in fact be reflecting the *T*. *testudinum* demography sensitivity to changes in both salinity gradient and mangrove discharges (e.g. CDOM and inorganic nutrients) triggered by the rainfalls arrival. Consequently, it is reasonable to expect that such a population structure response might be amplified under events associated with extreme precipitation in the region.

Peak *T*. *testudinum* shoots in the brackish inner bay during the rainy season of 2007 may be connected with the passage of Hurricane Dean in the southern Sian Ka’an Biosphere Reserve on 21 August 2007 (less than two months before the rainy season survey was undertaken), which accounted for about 10% of average annual precipitation and inflicted also significant damage on the vegetation of the region [47]. The remineralization of denuded organic matter following the hurricane landfall was likely high. This process might be associated with the strong correlation (r = -0.93; p-value = 0.01) recorded between ammonium and salinity in the southern-most bay where most of the freshwater input takes place.

Thus, the sudden increase in shoot number recorded in the SW subsystem and central basin in the aftermath of Hurricane Dean is possibly a consequence of both high water-borne nutrient pulsed to the watershed following the storm and rapid colonization by young shoots across open spaces left in the substrate due to wind shear. However, shoot density increase was muted toward the bay’s seaward endmember (Fig 9b), which may be a result of both a sheltered condition in the inner bay and freshwater flux attenuation as it gets closer to the bay’s ocean boundary [23]. This pattern contrasts with a dramatic decrease in shoot density of *T*. *testudinum* and slow recovery observed in the west coast of Florida as a consequence of atypically high precipitation during a strong El Niño event [48].

Even in the absence of extreme meteorological conditions (i.e., 2006 survey), inorganic nutrients and dissolved organic matter delivered to the bay during the onset of the rainy season, when accumulated material during the preceding period is flushed out, may have stimulated new shoot recruitment in the inner-most bay (Fig 9b). The prominent role that these discharges play on SAV metabolism is exemplified in the relationship between the salinity gradient (along with nitrate plus silicate, both inorganic nutrients significantly supplied by freshwater input) and both aboveground biomass and shoot density portrayed by the RDA during the dry season 2006 (Fig 10).

Increases in shoot density during the rainy season were coupled with sharp reductions in total abundance (Fig 9a and 9b), as newly recruited individuals were smaller in length and width compared to those present in the dry season. However, all sampling stations also had a sharp decrease in the above/belowground ratio, suggesting that the belowground biomass was stable and less affected by seasonal changes than the aboveground component (Fig 7c). This pattern agrees well with that observed in the sub-tropical coastal lagoon Lower Laguna Madre, where belowground biomass of *T*. *testudinum* lacks a seasonal pattern [42].

It is possible that the variability exhibited by *T*. *testudinum* among sites is a function of the frequency and magnitude at which environmental pressure events occur. For instance, exogenous nutrients and organic material supply to the inner embayment allows development of high biomass stands under low salinity conditions. Also, the relatively shorter and narrower blades observed on *T*. *testudinum* individuals growing in the brackish embayment compared to those in the marine-influenced mangrove key (Fig 8) is consistent with the inverse relationship between *T*. *testudinum* leaves width and freshwater observed in Biscayne Bay, Florida [18]. Additionally, high leaf asymmetry (skewness >1) exhibited in the central bay during rainy period (Fig 8a) may be a consequence of short-term salinity variability induced by strong tidal mixing between marine water and nutrients-enriched, brackish water masses in this site [49].

Another adaptation in *T*. *testudinum* to a stochastic, storm-influenced setting is the response of vertical elongation to burial events. While lateral rhizome lengthening is variable ranging from as little as 10 cm per year [50] to 55 cm per year [42] and rates tend to be ten-fold higher than vertical expansion, vertical shoot growth displays a more cyclic behavior associated with annual phenology (seasonal-scale), and spatial patterns regulated by varying energy conditions [51]. Likewise, the interannual variability in vertical growth adds to the ecological resilience of this species as documented after two strong hurricanes made landfall in a northern area from BA. Enhanced vertical growth rates of *T*. *testudinum* counteracting burial of leaf-producing basal meristems affected by heavy sediment deposition were found during Hurricane "Gilbert" (category-5 in September 1988) [51] and Hurricane "Wilma" (category-4 in October 2005), allowing recovery to levels prior to such events [37].

Recent global climate projections point out increases in average precipitation and heavier rainfall events under a warmer scenario [52]. Therefore salinity and inorganic nutrient variability will be increasingly tied to enhanced freshwater input to coastal zones. Seagrass communities developing along coastal waters should display effective mechanisms at distinct levels of organization (physiological, structural) to adjust to long-term continuous changes in the ambient and prevail under emergent conditions [53]. Awareness of potential responses in coastal habitats to this global pattern is particularly important in the Mexican Caribbean (eastern Yucatan Peninsula), where the aquifer constitutes a pathway for nutrients and pollutants delivery into the coastal zone [54].

The control of precipitation on the chemical composition of *T*. *testudinum* leaves in this region has been show in two ecosystems north of the study site. Peak phosphorus concentrations recorded in leaf tissue are related to heavy rainfall events [55], and trace metals enrichment was determined following hurricanes passage across the zone [56]. The intensity of cyclone activity has increased in the North Atlantic since 70’s and arguably correlates with warming scenarios from current climate models [22]. Expectations are that tropical cyclones will be 2–11% more intense by the end of the 21^st^ century. Such a pattern will be reflected both in increases of roughly one Saffir-Simpson category and 20% precipitation brought about by such atmospheric processes [57].

Groundwater discharges in coastal Yucatan are likely to be important to seagrass nutrition and health [54, 55, 58–60]. High nitrate and silicate concentrations measured in coastal aquifer discharges of the northern Yucatan Peninsula (NO_3_^-^ = 84.92 ± 5.88 μM, SRSi = 117.90 ± 11.00 μM) [27] suggests these physiographic features have a potential to deliver significant nutrient to the root zone of SAV communities in the region. The inverse relationship between the salinity gradient and SRSi concentrations both in dry (r = -0.85; p-value = 0.01) and the rainy seasons (r = -0.71; p-value = 0.01) supports the hypothesis that groundwater represents an important external contribution of this nutrient to the system.

The spatial distribution of CDOM during the dry season indicates that external input from adjacent wetland via surface waterways is prevalent, also suggesting that relatively high biomass of *T*. *testudinum* in the brackish inner bay during dry season might be explained by import of nutrients and organic material from neighboring ecosystems. Thus, *T*. *testudinum* beds growing landward may benefit seasonally from watershed organic material discharged into the system. The relevance of such loading is consistent with the dramatic drop of CDOM concentrations as a function of distance from the mangrove shoreline, with a sharp concentration drop crossing the channel towards the main bay (Fig 5d).

The responses exhibited by the seagrass *T*. *testudinum* in the bay, particularly those beds located in the proximity of the system’s terrestrial margin, illustrate a relatively higher resilience to changes in the watershed features relative to stands developing in more buffered environments of the system. These traits provide insights on the adaptation capacity of this species to increasingly variable climatic events.

## Supporting Information

S1 Fig Detrended Correspondence Analysis (DCA) biplot depicting the first two axes. The length gradient in the first axis = 1.037 and second axis = 0.797 are consistent with a linear (e.g., short gradient) instead of a unimodal relationship among seagrass variables.
(TIF)

S1 Table Log file of the DCA run with the biological set. These results supported the decision to implement a RDA to analyze relevant relationships between environmental and biologic variables.
(LOG)
