# Supplementary figures and images for: Response of Thalassia Testudinum Morphometry and Distribution to Environmental Drivers in a Pristine Tropical Lagoon
Source: PLoS One. 2016 Oct 13;11(10):e0164014. doi: 10.1371/journal.pone.0164014 (PMC5063390; doi:10.1371/journal.pone.0164014)

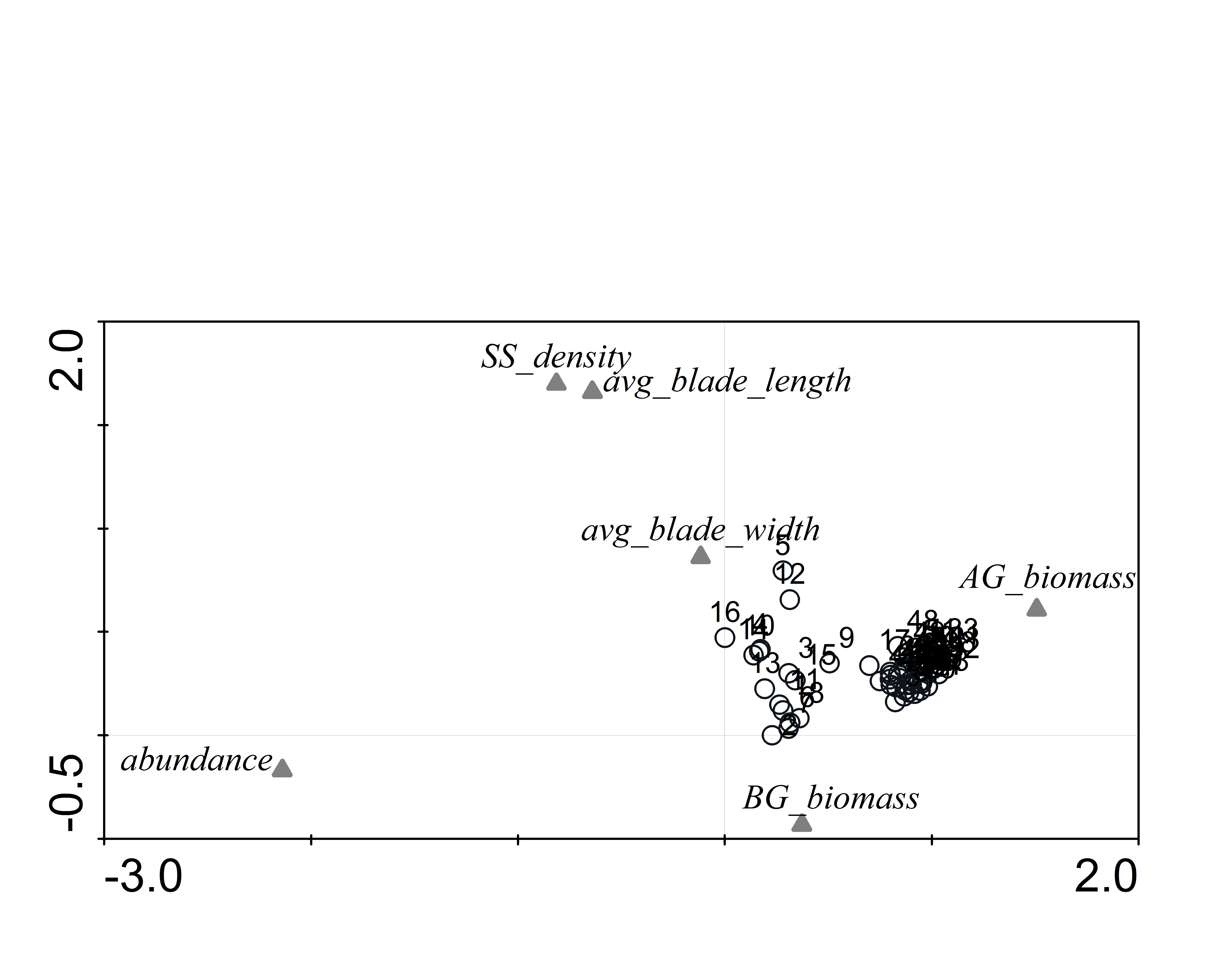

Supplement: S1 Fig — The length gradient in the first axis = 1.037 and second axis = 0.797 are consistent with a linear (e.g., short gradient) instead of a unimodal relationship among seagrass variables. (TIF) [file pone.0164014.s001.tif]
